# Smart Shoe-Assisted Evaluation of Using a Single Trunk/Pocket-Worn Accelerometer to Detect Gait Phases †

**DOI:** 10.3390/s18113811

**Published:** 2018-11-07

**Authors:** Marco Avvenuti, Nicola Carbonaro, Mario G. C. A. Cimino, Guglielmo Cola, Alessandro Tognetti, Gigliola Vaglini

**Affiliations:** 1 Dipartimento di Ingegneria dell’Informazione, University of Pisa, Largo Lazzarino 1, 56122 Pisa, Italy; marco.avvenuti@unipi.it (M.A.); nicola.carbonaro@unipi.it (N.C.); mario.cimino@unipi.it (M.G.C.A.C.); gigliola.vaglini@unipi.it (G.V.); 2 Research Center “E. Piaggio”, University of Pisa, Largo Lucio Lazzarino 1, 56122 Pisa, Italy

**Keywords:** accelerometer, foot contact detection, gait analysis, gait phase detection, pocket-worn, smart shoe, wearable sensor

## Abstract

Wearable sensors may enable the continuous monitoring of gait out of the clinic without requiring supervised tests and costly equipment. This paper investigates the use of a single wearable accelerometer to detect foot contact times and estimate temporal gait parameters (stride time, swing and stance duration). The experiments considered two possible body positions for the accelerometer: over the lower trunk and inside a trouser pocket. The latter approach could be implemented using a common smartphone. Notably, during the experiments, the ground truth was obtained by using a pair of sensorized shoes. Unlike ambient sensors and camera-based systems, sensorized shoes enable the evaluation of body-worn sensors even during longer walks. Experiments showed that both trunk and pocket positions achieved promising results in estimating gait parameters, with a mean absolute error below 50 ms.

## 1. Introduction

Spatio-temporal gait parameters can reveal important information related to health and well-being. For instance, some studies have shown that abnormal gait is linked with a higher risk of falling, and gait analysis has been proposed for automated fall-risk assessment [1,2,3]. Other works have reported that a deviation in gait patterns can be an early indicator of cognitive impairment caused by a neurodegenerative disease [4,5,6]. Furthermore, it has been demonstrated that some gait parameters are highly sensitive for the identification of the frailty syndrome, which is characterized by reduced strength and motor ability [7,8,9,10].

In recent years, there has been a significant effort in the development of automated techniques to help clinicians achieve a more objective assessment of gait in a controlled environment. Optoeletronic systems and force platforms represent the state-of-the-art in this field. Nevertheless, these techniques typically require a relatively complicated setup, based on costly equipment and/or multiple inertial sensors. As a consequence, subjects are still required to visit a clinic and perform predefined tests under the supervision of an expert. Wearable sensor-based systems have also been studied as a potential alternative for the analysis of gait parameters [11,12,13,14,15], especially when the target is the assessment in real-life conditions. The majority of wearable solutions relied on multiple tri-axial inertial measurements units (IMUs)—i.e., combinations of accelerometers, gyroscopes and, in some cases, magnetometers—applied to different lower body segments (thigh, shank, foot). The pioneering work of Jasiewicz et al. [16] demonstrated that foot- and shank-mounted inertial units were comparable with foot switches in detecting toe-off and heel-strike events. As a second relevant example, Lau et al. [17] employed a system of accelerometers and gyroscopes attached at the thigh, shank, and foot of a single leg and evaluated the reliability of different threshold-based methods for the detection of gait events on subjects with dropped foot and controls.

This paper aims to investigate the capability of a *single* wearable sensor to continuously monitor gait parameters out of the clinic. The motivation is that the use of a single device is key to obtaining an unobtrusive solution and fostering user acceptance in the real world. The contribution is to demonstrate that a single accelerometer can be used to estimate temporal parameters, such as the duration of gait cycles and the duration of phases within a cycle, during unconstrained walks. Hence, it would be possible to constantly monitor the trend of gait parameters over time, enabling early detection of changes that may require clinical attention. Unlike the examples described in Reference [16,17], our work employs a *single* tri-axial accelerometer for the detection of gait events—*heel-strike* (HS) and *toe-off* (TO) as initial and final foot contact during gait—and the consequent estimation of the duration of gait cycles (stride time) and gait phases (stance and swing duration). We considered and compared two alternative body positions for the sensing device: over the lower trunk (close to the L3 vertebra) and inside a front trouser pocket. To the best of our knowledge, the use of a device placed inside a trouser pocket (a common position for smartphones) has not been explored in previous studies. Notably, the results obtained were compared with the ground truth provided by a pair of sensorized shoes. Unlike standard reference systems based on optoelectronics instruments or force platforms, the use of sensorized shoes as reference enables gait analysis even during longer and unconstrained walks. Therefore, the proposed experiments represent a first step towards the evaluation of wearable-based gait analysis in free-living conditions.

The proposed technique for gait analysis with a sensor over the trunk was inspired by the techniques presented in previous works [18,19]. The lower back is a convenient body location to analyze acceleration patterns, as it is close to the body’s center of mass. Differently, a sensor placed in a pocket is subject to spurious accelerations during gait, resulting in a more challenging signal processing task. On the other hand, this approach could pave the way for the use of smartphones, which are often carried in a trouser pocket, as a novel means to perform basic gait analysis out of the clinic.

This article is an extension of the preliminary study we presented at the EAI MobiHealth 2017 conference [20].

## 2. Related Work

Wearable sensor-based systems have been proposed as a convenient tool to monitor gait and other activities in uncontrolled environments [21,22,23,24], so as to enable pervasive healthcare [25]. In particular, researchers have explored the use of wearable accelerometers to reliably detect foot contact times within gait cycles and estimate spatio-temporal gait parameters. In this context, a commonly adopted trade-off between accuracy and usability is represented by placing a single wearable accelerometer over the lower trunk. Zijlstra and Hof performed pioneering work in this field [18,26]. In particular, they proposed modeling the body’s center of mass trajectory during walk as an inverted pendulum. This model can be exploited to estimate heel-strike (HS) events by analyzing the anterior-posterior (AP) acceleration. The AP signal is first low-pass filtered at 20 Hz and 2 Hz. Then, the peak in the 20 Hz signal preceding a positive-to-negative transition in the 2 Hz signal is used to find HS events. Toe-off (TO) events were not considered in these experiments. In recent years, this method has been evaluated by different authors, and applied to healthy [27] as well as to pathological gaits [4].

The work presented in Reference [19] was among the first to explore the use of a single accelerometer positioned over the trunk to detect not only HS, but also TO events. Several empirical rules are used to detect HS events as a local maximum in the AP signal, whereas the respective TO is found by searching in vertical acceleration for the first local minimum after the HS event. Reported average error was around 10 ms.

An interesting evaluation of five different methods [18,19,28,29,30] for the estimation of gait parameters with a single accelerometer over the trunk is presented in Reference [31]. The methods were tested by five volunteers: the ground truth in terms of foot contact times was found by means of a stereophotogrammetric system and two force platforms. Detection sensitivity (i.e., the proportion of real foot contacts that were detected) ranged from 80% to 100%, whereas the average error in the estimation of temporal gait parameters ranged from 10 to 70 ms.

A more recent work [32] evaluated the use of a smartphone’s accelerometer to detect foot contacts. However, like in the works described above, the device was placed over the trunk.

An approach based on sensorized insoles to validate body-worn accelerometers is found in Reference [33]. The force sensors embedded in the insoles were used to validate foot contact detection with an ear-worn accelerometer. The insoles communicated with a controller, which was placed around the volunteers’ waist. Results in estimating contact times during a 15 m walk in a corridor showed an absolute error of ∼35 ms and ∼73 ms for HS and TO, respectively.

As previously mentioned, the majority of studies from the literature relied on ambient sensors to provide the ground truth, such as optoelectronic systems. This approach provides high accuracy, but does not enable the evaluation of foot contact detection during longer and less constrained walks.

## 3. Materials and Methods

**Figure 1:**
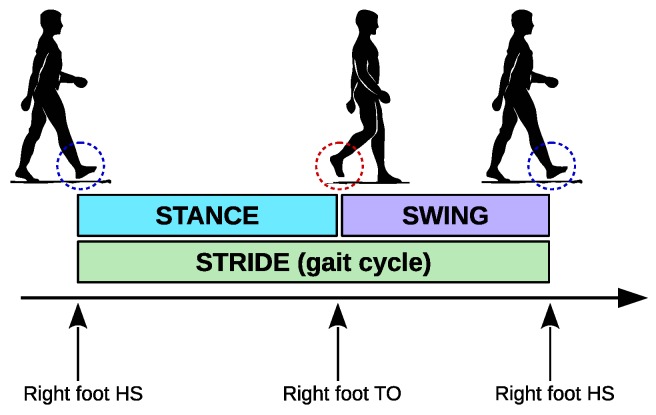
Gait cycle and its phases. A gait cycle is defined as the interval between consecutive heel-strike (HS) events of the same leg. The duration of a gait cycle is also known as *stride time*. The toe-off (TO) event defines the two phases of a leg during a gait cycle: stance (foot on the ground), and swing (foot swinging after toe-off and before heel-strike).

The proposed techniques aim to detect foot contacts in order to estimate the main temporal characteristics of gait cycles: the stride time and the duration of the stance and swing phases. A *gait cycle* is defined as the interval between two consecutive *heel-strike* (HS) events of the same foot (Figure 1). The duration of a gait cycle is known as *stride time*. Gait is further characterized by the instants at which a foot leaves the ground and starts “swinging” forward: this is known as a *toe-off* (TO) event (some works refer to heel-strike and toe-off as initial foot contact and final foot contact, respectively). For each leg, a gait cycle is characterized by two phases: *stance* (leg support, lasting from HS to TO) and *swing* (leg swinging forward, lasting from TO to the next HS).

**Figure 2:**
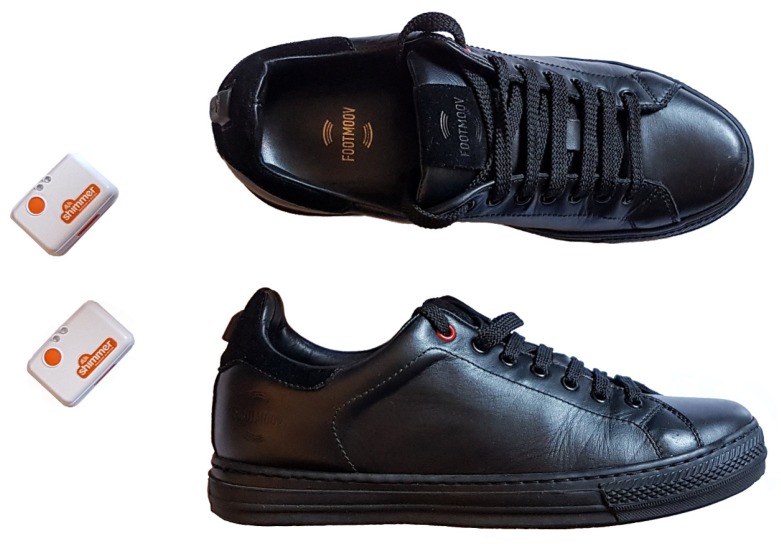
Shimmer3 devices (**left**) and FootMoov 2.0 shoes (**right**).

The ground truth for our experiments was based on a pair of FootMoov 2.0 shoes and two Shimmer3 devices, shown in Figure 2. FootMoov 2.0 (manufactured by Carlos s.r.l., Fucecchio, Italy) is a new version of the smart shoe described in Reference [34]. Five pressure sensors are integrated under the insole to monitor the mechanical interaction of the foot with the ground. Three of the sensors are positioned under the forefoot, while the remaining two are under the heel. These sensors are custom-made piezoresistive transducers produced by using ink-jet printing of a conductive material on a flexible substrate. Force sensors are sampled at 50 Hz. The Bluetooth 4.0 transmission module, fully integrated with the rest of the electronic unit in the heel of the shoe, enables low-energy data transmission to a mobile device (smartphone, tablet).

As described in the Introduction, we studied the use of a single tri-axial accelerometer worn at two alternative body positions (i.e., lower trunk and trouser pocket) to estimate stride time and stance and swing duration. In particular, we employed Shimmer3 devices (manufactured by Shimmer Sensing [35], Dublin, Ireland), which embed an ST Micro tri-axial accelerometer. The accelerometer was set to operate with 200 Hz sampling frequency and within a ±8 g range.

**Figure 3:**
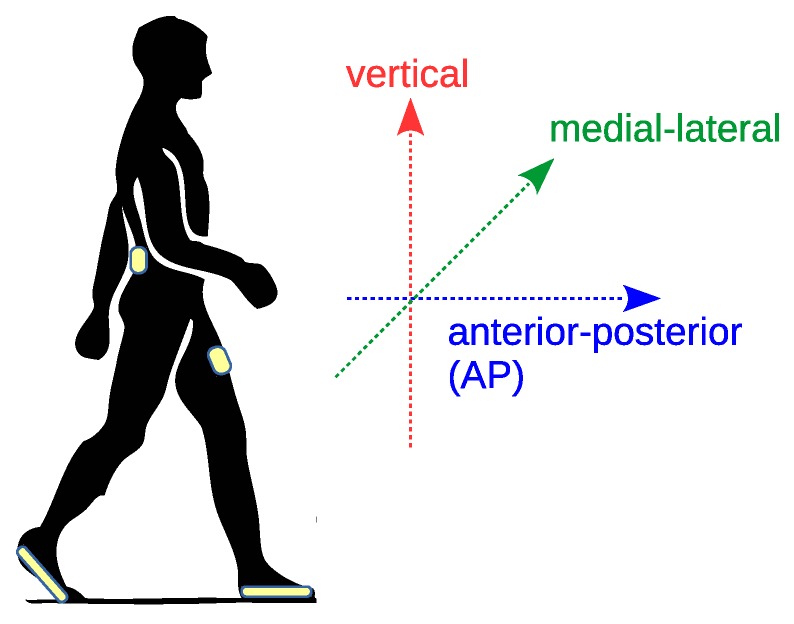
Device placement during experiments (Shimmer3 at trunk and pocket positions; FootMoov 2.0 shoes) and reference anatomical directions. In particular, the anterior-posterior (AP) direction is aligned with the direction of motion during gait.

Device placement is described in Figure 3, which also shows the anatomical directional references (vertical, anterior-posterior AP, and medial-lateral directions). Shimmer3 devices were positioned over the lower trunk (close to the L3 vertebra) and in a front trouser pocket (on the thigh). Hereafter, we refer to the two Shimmer devices as *trunk* and *pocket* sensors, respectively. We fixed the trunk device with an elastic band in an attempt to align the sensor reference frame (x,y,z) with the anatomical directional references. In particular, we aligned the *z* axis with the AP direction. The pocket sensor was not firmly fixed, the only precaution was to adopt the same convention when the user is standing still (i.e., *z* axis aligned with the AP direction as much as possible). Note that we have not applied any calibration procedure (e.g., using the technique described in Reference [36]) to align the sensor frame with the anatomical directions. This is further discussed in Section 5. Hereafter, for the sake of simplicity, we refer to the acceleration measured on the *z* axis as AP acceleration.

In the following subsections, we first describe the algorithm used to estimate gait parameters using the sensorized shoes. Then, we describe how the same parameters are estimated using the trunk and pocket sensors. Finally, we describe the experimental procedure.

### 3.1. Sensorized Shoe Method

**Figure 4:**
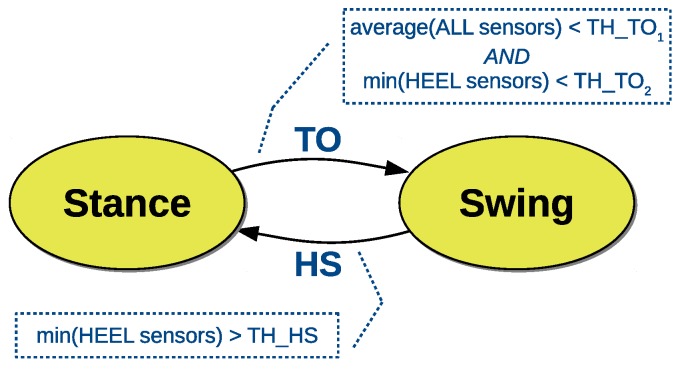
Detection of gait phases with the sensorized shoe. The thresholds TH_TO_1_ and TH_TO_2_ are used to detect a transition from stance to swing (TO event). A single threshold TH_HS is used to detect a transition from swing to stance (HS event). Thresholds were determined experimentally.

The algorithm to detect foot contacts is described by the finite state machine in Figure 4. A TO event determines a transition into the swing state and is detected when two conditions occur: (i) the average of all force sensors is below a threshold (TH_TO_1_); (ii) the minimum force value on the heel is below a threshold (TH_TO_2_). Conversely, a HS event leads to the stance state and is detected when the minimum force value on the heel is above a threshold (TH_HS). Thresholds were determined on separate experiments by using a high-frequency video trace (240 fps) as reference. According to our experiments the estimation accuracy of the shoe-based method is mostly limited by the sampling frequency of the in-shoe electronics (50 Hz), showing a mean error of ∼20 ms.

**Figure 5:**
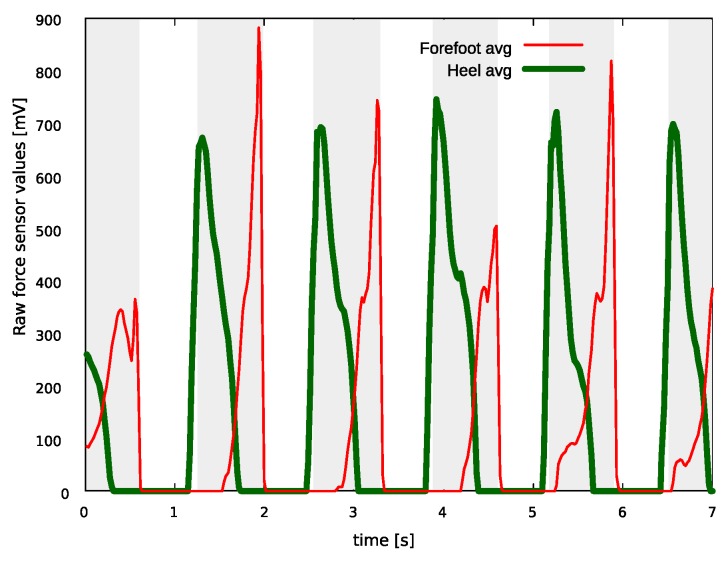
Example of force sensor signals on a FootMoov shoe during gait. Shaded areas highlight stance intervals.

An example of force sensor signals during gait is shown in Figure 5. For better clarity, signals at the heel (thick green line) and forefoot position (red line) are merged using the average value of the respective force sensors. The gait pattern is clearly visible (stance intervals are highlighted by the shaded areas). At the beginning of this example, the foot is in the stance state, as both heel and forefoot show non-zero values. Shortly after, there is a peak in the forefoot signal, showing that the weight is entirely loaded on the forefoot and that the foot is about to leave the ground. In fact, immediately after this peak all sensor values are close to zero, indicating a transition to the swing state. Then, as expected, a new HS event occurs, highlighted by a peak in the average heel signal, and the foot returns to the stance state.

The proposed algorithm is an enhanced version of the technique we presented in Reference [20]. Instead of using single force sensor values, the new algorithm relies on two simple features: the average of all force values and the minimum force value at the heel position. This makes detection more robust in the scenario where one of the sensors shows values slightly greater than zero during the swing phase (for instance, because of calibration errors).

### 3.2. Trunk Sensor Method

The method to find temporal gait parameters at the trunk position proceeds as follows. First, gait cycles are identified using the walking detection algorithm presented in Reference [37]. Walking detection exploits a threshold (determined experimentally) to find groups of peaks in the acceleration magnitude signal (Euclidean norm). Each group is produced by foot contact with the ground after the swing phase and at the beginning of the stance phase. The algorithm looks for gait segments made of at least eight steps, and a segment ends when an interval of 1 s without further steps is found. The reader is forwarded to Reference [37] for more details on this technique, as the focus is here on the detection of gait phases.

The interval including the group of acceleration peaks produced at each step is then used to search for HS and TO events in the AP signal. More precisely, HS events correspond to a local maximum, whereas TO events correspond to a local minimum in AP acceleration.

**Figure 6:**
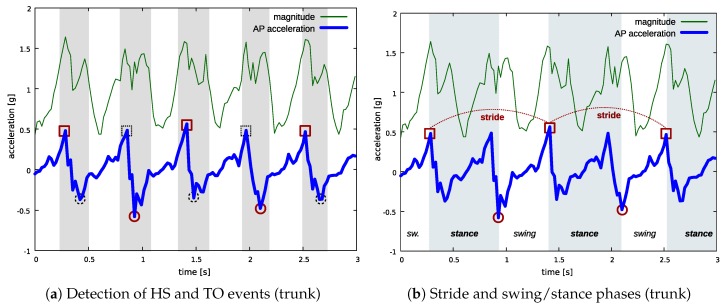
Detection of gait parameters at the trunk position. In (**a**): shaded areas highlight the intervals identified by the walking detection algorithm based on acceleration magnitude analysis; squares indicate HS events, circles indicate TO events; red color is used for the events of one leg, black dashed for the other one. In (**b**) the estimated parameters are shown for the leg making the first step in this example: shaded areas highlight stance periods.

Figure 6a,b show how the proposed approach is applied to an acceleration pattern including two full gait cycles (for the leg making the first step) and five steps in total. The thin green line is the acceleration magnitude, whereas the thick blue line is the AP acceleration. In Figure 6a, shaded areas highlight the intervals defined by means of the groups of peaks in the acceleration magnitude signal (steps): HS and TO events are found within these intervals. For the leg making the first step, red squares and circles show the detected HS and TO events, respectively, whereas black dashed squares and circles show HS and TO events for the other leg. Finally, Figure 6b shows the same gait pattern and highlights the estimated gait parameters for the leg making the first step in this example (stride time, swing, and stance).

### 3.3. Pocket Sensor Method

As in the trunk method, the walking detection algorithm described in Reference [37] is applied to the acceleration magnitude signal to find the intervals including HS and TO events (steps). However, in the pocket method, HS and TO events are found only for the leg that is carrying the sensor. Hereafter, we refer to the steps made with the leg that is carrying the sensor as *sensor steps*. AP acceleration is analyzed to discriminate between sensor steps and the steps made with the contralateral leg: sensor steps show an average AP acceleration above a threshold determined experimentally. Sensor steps are used to detect HS events by finding the local maximum value on AP acceleration, whereas contralateral steps are used to detect TO events by finding the local minimum on AP acceleration.

**Figure 7:**
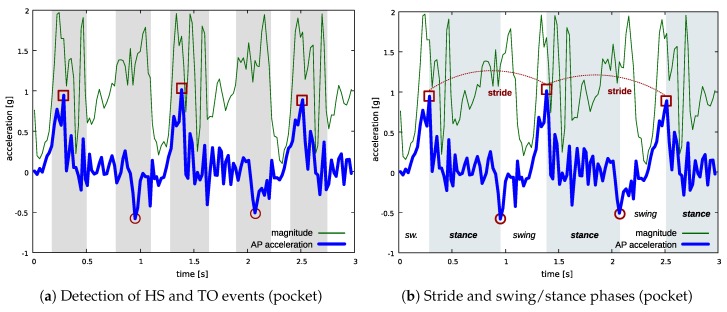
Detection of gait parameters at the pocket position. In (**a**): shaded areas highlight the intervals identified by the walking detection algorithm based on acceleration magnitude analysis (sensor steps and contralateral steps); red squares indicate HS events, whereas red circles indicate TO events—both events are detected only for the leg which is carrying the sensor. In (**b**) the estimated parameters are shown: shaded areas highlight stance periods.

The example in Figure 7 shows how the method is applied to the acceleration pattern measured at pocket position during the same gait cycles as in the trunk example described in Figure 6. The green thin line is the acceleration magnitude signal, whereas the blue thick line is the acceleration measured on the axis aligned with the AP direction when the user is standing still. For the sake of simplicity, we refer to this signal as AP acceleration as in the trunk experiment. Shaded areas in Figure 7a highlight the intervals identified by the walking detection algorithm (sensor steps and contralateral steps). Red squares indicate the HS events detected during sensor steps, whereas red circles indicate the TO events. Figure 7b shows the estimated gait parameters for the leg which is carrying the sensor, in terms of stride times and swing/stance phases.

### 3.4. Procedures

Three volunteers were recruited for data collection: their main characteristics are shown in Table 1. User 1’s gait was potentially impaired, as he had a plaster cast on his left arm (due to forearm fracture). Volunteers wore a pair of sensorized shoes and the two Shimmer sensors (trunk and pocket) during the experiments, as described in Figure 3. The experiments consisted of performing two straight walks of about 40 m on level ground. Video traces were collected during the experiments. Shimmer acceleration samples were downsampled to 50 Hz to obtain the same sampling frequency used by the shoes. Collected samples were stored on persistent memory to ensure repeatable evaluation.

The force sensor signals on the shoes were used to find stride time and the duration of stance and swing for each gait cycle and for each foot. The same parameters were estimated at the trunk and pocket position using the respective methods and acceleration signals, enabling direct comparison with the shoe-based ground truth.

As part of the procedure, we checked that all the methods detected the correct number of foot contacts (HS and TO events). This was verified by comparing the results with the video traces, which also allowed us to verify that no “false steps” were detected and no real steps were missed at the beginning and at the end of walks, when movements are typically less regular.

## 4. Results

Table 2 shows the estimated gait parameters at the shoe, trunk, and pocket position for all the users. The table shows the mean and standard deviation values calculated over all the gait cycles, which were performed in two separate walks. Times are expressed in seconds and were rounded to the nearest tenth of a second. For shoe and trunk, results related to left and right legs were merged as the recognition of a specific side is beyond the scope of this study. In addition, all the users showed a symmetric gait (the difference between legs was below 20 ms for all parameters). In each walk, initial and ending gait cycles were removed as they tend to be highly irregular. It is immediately evident from Table 2 that User 1 walked with a substantially lower cadence during these experiments with respect to the other users (a cycle took 1.33 s on average for User 1, compared to about 1.1 s for User 2 and User 3).

A first comparison of the results obtained through different approaches is contained in Table 3, which shows the mean absolute error (MAE) of Shimmer-based estimations with respect to shoe-based estimations.

**Figure 8:**
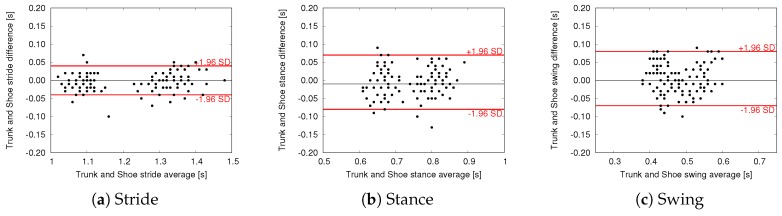
Bland–Altman plots—Trunk vs. Shoe measurements.

**Figure 9:**
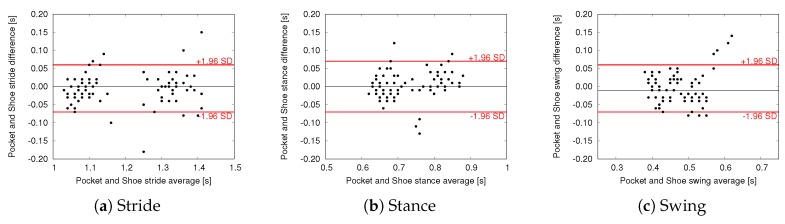
Bland–Altman plots—Pocket vs. Shoe measurements.

To further evaluate the agreement between estimations we performed Bland–Altman (BA) analysis, where the difference of two paired measurements is plotted against the mean of the two measurements. BA plots comparing trunk and pocket estimations with shoe-based estimations are shown in Figure 8 and Figure 9, respectively. In these plots the black line represents the mean difference between the two methods (i.e., the mean error committed by the proposed Shimmer-based method with respect to the shoe-based reference), whereas the red lines indicate the limits of agreement (LoA, found as mean difference ± 1.96 SD). Mean difference and LoA values are also reported in Table 4 and Table 5.

## 5. Discussion

Despite using simple algorithms, the proposed methods were able to detect foot contacts without producing false positives. This is an essential requirement for accurate gait analysis. A glimpse at the results in Table 2 confirms that gait parameters can be usefully estimated using a single accelerometer, as both trunk- and pocket-based methods managed to capture the same gait characteristics as done by the shoe-based method. Notably, as observed in Section 4, User 1 performed the experiment with a substantially different pace: this was probably due to the presence of the plaster cast. As a result, these experiments enabled an evaluation of the proposed methods at different cadence.

A low estimation error is confirmed by the analysis of the MAE, presented in Table 3. In particular, the trunk sensor estimated stride times with less than 30 ms MAE, whereas stance and swing were estimated with a MAE of less than 50 ms. As expected, swing and stance estimations are relatively less accurate than stride, as they depend on accurate detection of both HS and TO events. These errors are comparable with the sampling period of the shoe force sensors (∼20 ms) and in line with the best performing systems reported in Reference [31]. As far as a comparison between trunk and pocket approaches, stride estimation by a pocket sensor shows a slightly higher MAE, but this did not affect the correctness of average estimations. Stance and swing estimations show similar results as in the trunk experiment for User 1 and User 2, whereas for User 3 the in-pocket approach provides better results (i.e., lower MAE).

Further evidence of the agreement between Shimmer-based and shoe-based estimations is provided by the BA analysis. All the plots show that errors are randomly scattered within the range of agreement, without suggesting a clear relationship with the magnitude of the estimated parameter. In other words, according to our experiments, estimation accuracy is not influenced by walking characteristics such as cadence and stance/swing ratio. Indeed, the proposed methods obtained promising results on all subjects, despite their different pace, age, and health condition. The limits of agreement are in line with the errors reported by other works in the field of gait phase detection for IMU-based approaches [15,38], with the key difference that the proposed approach is based on lightweight algorithms and a single accelerometer.

BA plots also highlight the presence of a limited number of outliers, especially for the pocket approach, with some estimations showing an error in the order of 0.1 s. These sporadic errors can be explained considering that, inside a trouser pocket, the sensor is subject to more spurious accelerations during gait. For similar reasons, pocket also shows a relatively greater range of agreement for stride estimation. Nevertheless, the pocket approach managed to achieve similar results with respect to trunk in terms of mean difference, as shown in Table 4 and Table 5. More precisely, trunk and pocket achieved a mean error lower than 20 ms for all the parameters. It should be considered that, in the proposed application scenario, the method is used to evaluate the variation of mean values over time, rather than considering single gait cycles.

Overall, these results are very encouraging and support the hypothesis that an unobtrusive approach based on a single wearable sensor could be used to continuously monitor temporal gait parameters during everyday life. The use of a sensor carried in a trouser pocket is the most innovative aspect of our work. Despite the more challenging positioning (the orientation of the sensor changes during the swing phase), the results of this novel approach suggest that a pocket-worn device (like a smartphone) may be used to estimate mean gait parameters with high accuracy. We envision that such an unobtrusive approach could be used to continuously monitor some key gait parameters out of the clinic, enabling prompt detection of changes in gait patterns that may require medical attention. This could be of paramount importance for patients with a higher risk of developing conditions affecting motor ability, such as older adults. One specific example is frailty syndrome: in this context, early detection enables the adoption of preventive measures to reduce the impact of this chronic condition on the patient’s quality of life.

### Limitations

Some limitations of our work should be mentioned. First, we tested our algorithm only on level walking conditions, without considering typical situations such as walking on irregular ground or climbing a flight of stairs. To the purpose of monitoring the evolution of gait parameters in the long term, one possible solution could be to filter out these scenarios, for example exploiting a barometer to detect stairs and a filter based on autocorrelation to disregard gait on irregular terrain [23].

Another important limitation is the reduced number of subjects involved, and the fact that we did not involve patients with an impaired gait, such as frail subjects. However, this study was conceived as a preliminary evaluation of a simple and easy-to-use approach based on a single wearable sensor. The results related to the pocket approach, in particular, will enable extensive studies based on common smartphones. In future studies, we plan to significantly extend the number of subjects involved, and evaluate whether the use of personalized models could help further reduce estimation errors.

The pocket-based technique can only estimate the parameters related to the leg which is carrying the sensor. This does not represent a problem in symmetric gait, where both legs show similar parameters. Further experiments will be required to evaluate how to improve the algorithm to cope with patients with highly asymmetric gait. For example, previous works reported that double support (DS) duration is highly correlated with frailty [10]. DS is the part of the gait cycle characterized by double leg support: it can be easily derived from swing and stance values of a single leg when gait is symmetric (DS = stride−2∗swing), but will require further investigation in case of asymmetric gait.

Finally, in our method, it is supposed that one of the reference axes of the accelerometer is roughly aligned with the AP direction when the user is standing still. Despite this limitation, we still believe that our results are relevant. Indeed, because of its form factor, a smartphone is typically placed in a front trouser pocket with the screen orthogonal to the AP direction. Hence, the axis orthogonal to the screen is roughly aligned with AP during gait, as in our assumption. Additionally, it is promising that despite the lack of accurate calibration procedures our method managed to obtain low estimation errors. Nevertheless, in future works, the use of automated calibration techniques [36] will be key to reducing estimation errors and improving the robustness of the method, especially when the smartphone is carried in a different way.

## 6. Conclusions

In this work, we have evaluated the use of accelerometers placed at two different body positions, over the lower trunk and inside a trouser pocket, as a means to estimate temporal gait parameters. Notably, we relied on sensorized shoes to obtain the ground truth during experiments. This enabled the evaluation of longer walks with respect to ambient sensors like cameras or instrumented mats. Results are very promising and show that both approaches (trunk and pocket) are able to provide an accurate estimation of stride time and swing/stance phases. The method for a device placed inside a pocket is novel, and suggests that a common smartphone may be used as an unobtrusive solution to continuously monitor key gait parameters out of the clinic.

In future studies, we plan to perform extensive experiments to further investigate the use of common smartphones for gait analysis, possibly including older adults with impaired gait in the experiments. We also plan to perform similar tests with a wrist-worn device (like a smartwatch), which could represent a further step towards unobtrusiveness and ease of use. In the long term, we plan to apply these unobtrusive gait analysis methods to the detection and monitoring of medical conditions affecting gait, such as frailty. In this context, the wearable-based approach may significantly reduce the burden of these conditions on both patients and clinicians, by enabling continuous and automated monitoring of gait during everyday activities.

## Figures and Tables

**Table 1:** Volunteers’ characteristics.

ID	Age	Sex	Height [cm]	Weight [kg]	Notes
1	22	M	176	59	Injured arm.
2	32	M	175	62	N/A
3	61	F	166	66	N/A

**Table 2:** Estimated gait parameters (mean ± standard deviation).

**User 1**			
**Parameter**	**Shoe**	**Trunk**	**Pocket**
Stride time [s]	1.33 ± 0.04	1.33 ± 0.05	1.33 ± 0.05
Stance duration [s]	0.80 ± 0.03	0.80 ± 0.04	0.81 ± 0.05
Swing duration [s]	0.53 ± 0.03	0.53 ± 0.04	0.52 ± 0.05
**User 2**			
**Parameter**	**Shoe**	**Trunk**	**Pocket**
Stride time [s]	1.10 ± 0.02	1.10 ± 0.02	1.10 ± 0.03
Stance duration [s]	0.64 ± 0.01	0.66 ± 0.03	0.65 ± 0.03
Swing duration [s]	0.46 ± 0.02	0.43 ± 0.03	0.45 ± 0.03
**User 3**			
**Parameter**	**Shoe**	**Trunk**	**Pocket**
Stride time [s]	1.07 ± 0.03	1.07 ± 0.03	1.07 ± 0.03
Stance duration [s]	0.68 ± 0.02	0.64 ± 0.03	0.67 ± 0.02
Swing duration [s]	0.40 ± 0.01	0.43 ± 0.02	0.40 ± 0.01

**Table 3:** Mean absolute error (MAE) with respect to shoe-based estimations.

	User 1		User 2		User 3	
Parameter	Trunk	Pocket	Trunk	Pocket	Trunk	Pocket
Stride time [s]	0.02	0.03	0.01	0.03	0.01	0.01
Stance duration [s]	0.03	0.03	0.03	0.03	0.04	0.02
Swing duration [s]	0.03	0.04	0.03	0.03	0.04	0.01

**Table 4:** Results of Bland–Altman analysis—Trunk vs. Shoe.

Parameter	Mean Difference	LoA
Stride time [s]	0.00	−0.04 to 0.04
Stance duration [s]	−0.01	−0.08 to 0.07
Swing duration [s]	0.00	−0.07 to 0.08

**Table 5:** Results of Bland–Altman analysis—Pocket vs. Shoe.

Parameter	Mean Difference	LoA
Stride time [s]	0.00	−0.07 to 0.06
Stance duration [s]	0.00	−0.07 to 0.07
Swing duration [s]	−0.01	−0.07 to 0.06
